# Patient acceptance and implementation of micronutrient therapy in women with neurostress-related symptoms

**DOI:** 10.1007/s00404-026-08321-6

**Published:** 2026-02-04

**Authors:** Fanette Loviat, Elena Pavicic, Norman Bitterlich, Petra Stute

**Affiliations:** 1 Faculty of Medicine, University of Bern, Bern, Switzerland; 2 Department of Obstetrics and Gynecology, University Hospital of Bern, Bern, Switzerland; 3 Graduate School for Health Sciences, University of Bern, Bern, Switzerland; 4 Freelance Statistician, Chemnitz, Germany

**Keywords:** Micronutrients, Neurostress, Acceptance, Adherence

## Abstract

**Purpose:**

To evaluate the acceptance of micronutrient therapy in women with symptoms related to a pathological neurostress profile and to explore its association with patient-reported outcomes such as perceived efficacy, side effects, compliance, and perceived lack of information.

**Methods:**

This retrospective observational study included women aged ≥ 18 years who underwent neurostress testing followed by micronutrient therapy. The study was conducted at the Department of Obstetrics and Gynecology, University Hospital Bern. Patients were invited to complete the validated ACCEPT© questionnaire to assess their level of treatment acceptance. Descriptive statistics and non-parametric tests were applied, and correlations were analyzed using Spearman’s rho.

**Results:**

Eighty-one women participated. Overall treatment acceptance was high, with a median ACCEPT© score of 88.9 (Q1 = 66.6, Q3 = 100). Acceptance of drug-related constraints scored high (median = 100), whereas acceptance of treatment duration was lower (median = 66.6). Side effects were well tolerated (median = 100), and perceived efficacy was rated positively (median = 100). General acceptance correlated significantly with perceived efficacy (p < 0.001) and side effects (p < 0.001), but not with compliance (p = 0.084). A negative correlation was found with the perceived lack of information (p = 0.043).

**Conclusion:**

Micronutrient therapy in women with a pathological neurostress profile was highly accepted, particularly regarding treatment-related constraints and side effects. Acceptance was closely linked to perceived efficacy, highlighting the importance of patient-centered communication and expectation management.

**Supplementary Information:**

The online version contains supplementary material available at 10.1007/s00404-026-08321-6.

## What does this study adds to clinical work

**Table:** 

This study shows that high adherence to micronutrient therapy is achievable in patients with neurostress imbalances when treatment is perceived as acceptable and effective. It emphasizes the importance of addressing concerns about treatment duration and improving patient education to support ongoing engagement in care.	

## Introduction

Stress is an adaptive response of the entire organism to physical or emotional stimuli that threaten its homeostasis [1]. The term “neurostress” refers to phenomena described in the literature as neurobiological, neuroendocrine, and neuroinflammatory stress [2–4]. It encompasses the physiological mechanisms involved in the stress response, as well as the potential damage caused by prolonged exposure to stress [5]. Repeated activation of stress responses can lead to neuroendocrine imbalances and contribute to the development of various symptoms [4, 6, 7].

The neurostress profile is a diagnostic tool that evaluates a range of hormones, such as cortisol, and neurotransmitters, including catecholamines, which are integral to physiological stress responses [8, 9]. It should be noted that the concept of a “neurostress profile” is not currently recognized in the medical literature as a standardized diagnostic approach. However, the measurement of individual components, such as cortisol and catecholamines, is well-established for assessing activation of the sympathetic nervous system [8–11].

Abnormal fluctuations in these substances can lead to various symptoms, including depressive mood, anxiety, irritability, sleep disorders, lack of motivation, headaches, concentration difficulties, fatigue, disrupted eating patterns, loss of vitality, decreased libido, and reduced stress resilience [12].

Several studies suggest that supplementing certain micronutrients may regulate neurotransmitter and hormone levels involved in stress responses, potentially improving mood, reducing stress, enhancing sleep quality, and alleviating the symptoms mentioned above [13–15].

However, adherence to long-term treatments can significantly impact their effectiveness [16]. Numerous studies have examined the role of treatment adherence, particularly in chronic conditions such as diabetes or hemophilia [17–20]. Sidani et al. (2009) identified several key factors that influence a treatment’s acceptability to patients. Those factors are for example appropriateness, compatibility with personal lifestyle, efficacy, and minimal side effects [21, 22].

Our primary goal is to evaluate the degree of therapy acceptance in patients with symptoms related to neurostress imbalance. Additionally, we aim to incorporate some of the treatment characteristics identified by Sidani et al. into our study. We seek to determine whether a relationship can be established between patient acceptance, adherence to treatment, and its perceived effectiveness.

This study was designed as a service evaluation and implementation-focused investigation of patient acceptance in routine clinical practice. It was not intended to assess the clinical effectiveness of either the neurostress profile assessment or the subsequent individualized micronutrient therapy.

Our hypothesis was that the micronutrient therapy is well accepted by the population studied. Furthermore, we assumed that the more acceptable and easy-to-implement the treatment recommendations are, the higher the patient compliance will be and the more effective the therapy will appear to be.

## Methods

### Study design

This was a single-center, retrospective observational study conducted at the Department of Obstetrics and Gynecology, University Hospital Bern, Switzerland, between November 2021 and March 2022. The study protocol was approved by the Cantonal Ethics Committee of Bern (Project-ID: 2021–01019) (Supplementary File 2). Written informed consent was obtained from all participants prior to inclusion.

### Study population

Eligible participants were German-speaking women aged ≥ 18 years residing in Switzerland who had undergone neurostress testing at the University Hospital Bern and subsequently received a micronutrient therapy. The neurostress profile is not a standardized or universally validated diagnostic approach, but rather a locally implemented assessment tool used in routine care at the study center to guide individualized micronutrient recommendations. In the context of this study, it served as a pragmatic framework for clinical decision-making rather than as a diagnostic endpoint.

The specifications of the micronutrient therapy are presented in Table 1. Exclusion criteria were the inability to complete the questionnaire due to language barriers, psychological disorders, or dementia; lack of internet access; and any form of coercion to participate.

**Table 1:** Therapy specification table

Substances utilized *Not all listed substances were administered to each participant. The composition was tailored to the individual clinical needs of the patients*	Vitamin B complex: Vitamin B1 (thiamine), vitamin B2 (riboflavin), vitamin B3 (niacin), vitamin B5 (pantothenic acid), vitamin B6 (pyridoxal-5-phosphate, pyridoxine), vitamin B12 (cobalamin), biotin, folic acid S-adenosyl-L-methionine (SAMe) 5-hydroxytryptophan Magnesium Zinc Alpha-lipoic acid Taurine Glycine *Rhodiola rosea root extract* L-glutamine L-carnitine L-tyrosine Coenzyme Q10 (ubiquinone) *Camellia sinensis leaf extract* L-phenylalanine Iron Copper *Mucuna pruriens seed extract*
Dosing ranges	Dosing ranges were individually determined
Number of daily units	One capsule per day
Duration of therapy	At least 3 months
Change over time	None
Therapy standardization vs. individualization	Composition and dosing ranges were individualized

### Setting

**Fig. 1:**
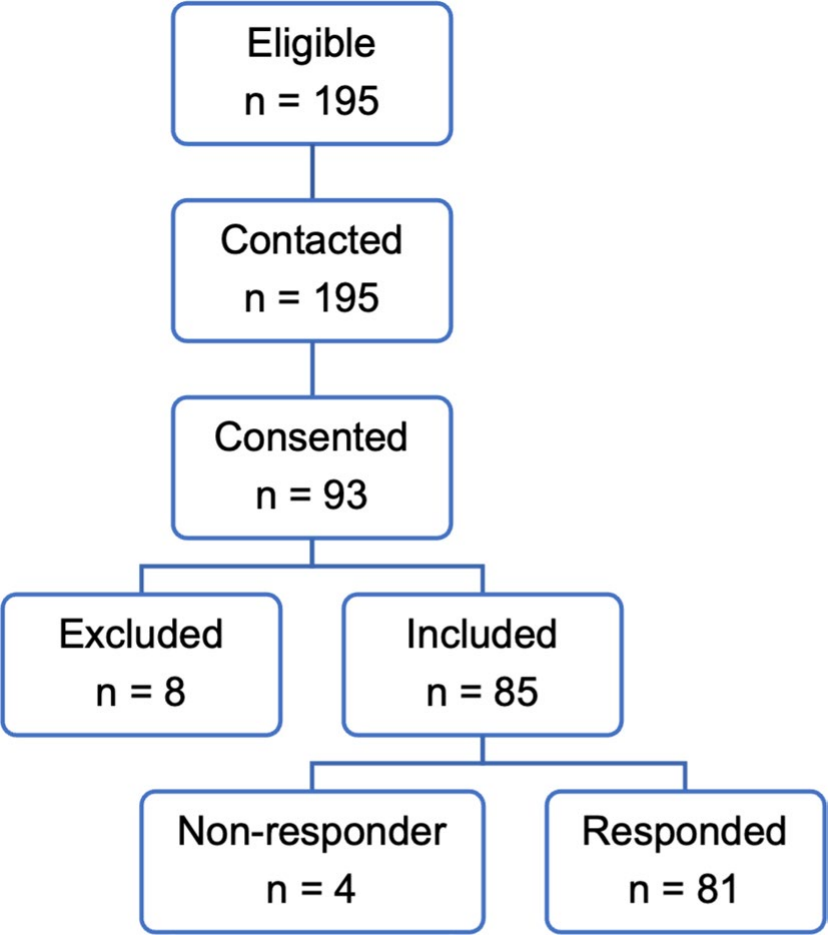
Flow diagram of study participants

Patients who met the inclusion criteria were contacted by postal mail by the researcher and invited to participate. After the signed informed consent was obtained, the participants received a hyperlink via e-mail with a personal login to the questionnaire (Fig. 1).

### Study instrument

Treatment acceptance was assessed using the validated ACCEPT© questionnaire, a generic 25-item instrument designed to evaluate how patients balance the advantages and disadvantages of long-term medication (Supplementary File 1). The questionnaire comprises six dimensions: acceptance of medication inconvenience, treatment duration, regimen constraints, numerous medications, side effects, and treatment effectiveness. In addition, three general items assess overall acceptance. Scores for multi-item dimensions were calculated from recoded responses according to the scoring manual and linearly transformed to a 0–100 scale, with higher scores indicating greater acceptance. The “numerous medications” dimension was scored on a 1–3 scale. Alongside the ACCEPT© questionnaire, demographic and clinical data were collected.

### Statistical analysis

According to the *User Manual of the ACCEPT© Questionnaire*, an expected effect size of at least 0.33 was anticipated. For the Wilcoxon test, used to compare a single sample against the constant value of 50, the minimum required sample size was calculated to be 77 participants. This calculation was based on a significance level of 5% and a statistical power of 80%.

Descriptive analysis was conducted on all collected data, including statistical measures such as mean, standard deviation, median, quartiles and range for continuous or ordinal variables. Non-parametric statistical tests were applied throughout the analysis.

### The questionnaire was analyzed according to the established protocol

Correlations between continuous variables were assessed using the non-parametric Spearman’s rho test. The 95% confidence intervals for the correlations were calculated using Fisher’s z transformation. Demographic variables were analyzed as pairwise comparison using the Mann–Whitney *U* test.

A p-value of less than 0.05 was considered statistically significant. The exploratory data analysis did not consider the influences of multiple testing for secondary outcomes. Missing data were noted as such and were not imputed.

Data were analyzed using SPSS software version 29.0.

## Results

A total of 81 patients agreed to participate in the study. The educational attainment, income and occupation rate correspond to the demographics of the population studied, according to 2024 demographic data from the Swiss Federal Statistical Office (FSO) [23]. Descriptive statistics did not reveal any significant differences between the demographic groups as described in Table 2. As shown in Table 3, the primary symptoms were fatigue (95%, n = 76), reduced stress tolerance (93.8%, n = 75), and loss of vitality (90%, n = 72). Other symptoms listed in Table 2 were also reported frequently, reflecting the presence of multiple co-occurring symptoms. However, as the survey did not assess the specific reasons for initiating therapy, no direct conclusion can be drawn regarding their role in treatment-seeking behavior.

**Table 2:** Baseline characteristics of the study population (N = 80)

Characteristic	Value
*Educational level, n* (%)	
Secondary school or below	0/64 (0%)
Vocational training	29/64 (45%)
Tertiary education	35/64 (55%)
*Employment status, n* (%)	
Employed (part-/full-time)	51/66 (77%)
Not employed/retired/disabled	15/66 (23%)
*Income, n* (%)	
< 5′000 CHF	28/65 (43%)
5′000–10′000 CHF	27/65 (42%)
> 10′000 CHF	9/65 (14%)
No personal income	10/65 (15%)
*Marital status, n* (%)	
Married/partnership	49/75 (65%)
Single/divorced/widowed	26/75 (35%)
*Previous therapies tried, n* (%)	
Yes	47/78 (60%)
No	31/78 (40%)

**Table 3:** Baseline symptoms/comorbidities (N = 80)

Domain	Symptom	n (%)
Fatigue and Vitality	Fatigue/exhaustion	76 (95.0%)
	Reduced stress tolerance/overwhelmed	75 (93.8%)
	Reduced recovery/vitality loss	72 (90.0%)
Neuropsychiatric	Anxiety/inner unrest	71 (88.7%)
	Depressive mood	67 (83.7%)
	Irritability / aggressiveness	60 (75.0%)
	Forgetfulness / concentration problems	65 (81.2%)
Sleep & Drive	Sleep disorder	71 (88.7%)
	Lack of drive	71 (88.7%)
Somatic	Disordered eating /digestive problems	60 (75.0%)
	Headache / “brain fog”	51 (63.7%)
Sexual health	Lack of libido	67 (83.7%)

Most participants (93.5%, n = 72) reported taking their medication at least 6 days a week. 88.8% (n = 71) of participants reported feeling sufficiently to very well informed about the aim of micronutrient therapy. 60% (n = 47) had already tried one or more other treatment for their complaints. These previous treatments were not perceived as effective in 55% (n = 27) of cases.

The ACCEPT© dimension scores were calculated using clusters of items of the ACCEPT© questionnaire. All items were given equal weight in the calculation of the scores. The scores ranged from 0 to 100 for the multi-item dimensions and from 1–3 for the single-item dimension. All the scores were calculated only if at least 50% of the items in the dimension were completed.

### Primary endpoint

The analysis of overall treatment acceptance revealed a median score of 88.9, with individual scores ranging from a minimum of 22.2 to a maximum of 100. The first quartile (Q1) was 66.6 and the third quartile (Q3) was 100. Notably, 7.5% (n = 5) of participants gave a score below 50. This indicates that, on average, participants reported a high level of acceptance, though there was some variability across the group.

### Secondary endpoints

Most participants found the therapy’s requirements easy to manage, as reflected by a median acceptance score of 100 (Q1 = 80, Q3 = 100) and a mean score of 89.5 regarding drug-related constraints. In contrast, the acceptance of treatment duration scored lower, with a median score of 66.6 (Q1 = 66.6, Q3 = 83.3), indicating more mixed feelings about the sustainability of the treatment over time.

The acceptance of constraints related to the therapy regimen itself was relatively approved with a mean score of 76.3, a median score of 90 (Q1 = 60, Q3 = 100). Acceptance of side effects scored particularly high, with a mean score of 90, a median score of 100 (Q1 = 100, Q3 = 100), suggesting that participants experienced few negative side effects or found them manageable. Finally, the perception of the treatment’s effectiveness was generally positive, with a mean score of 83, a median score of 100 (Q1 = 75, Q3 = 100) for acceptance of treatment efficacy.

### Correlations

Statistical analysis revealed several noteworthy correlations. A correlation (r = 0.498, 95% CI [0.23, 0.69], p < 0.001) was found between general treatment acceptance and the perceived efficacy of the therapy, indicating that higher acceptance was strongly associated with a belief in the therapy’s effectiveness.

In contrast, the correlation between general acceptance and compliance was weaker (r = 0.216, 95% CI -0.03 to 0.44) and not statistically significant (p = 0.084), suggesting that factors beyond general acceptance may play a larger role in determining compliance.

A modest negative correlation (r = -0.248, 95% CI [-0.46, -0.01], p = 0.043) was observed between general acceptance and the perceived lack of information, implying that participants who felt less informed were somewhat more likely to report lower treatment acceptance. Additionally, a significant correlation (r = 0.401, 95% CI [0.18, 0.59], p < 0.001) was found between general acceptance and the perception of side effects, showing that those who experienced fewer side effects were more likely to accept the treatment.

The Mann–Whitney *U* test did not reveal a significant difference in treatment general acceptance between the low-income and high-income groups (U = 396.5, Z = -1.62, p = 0.105). Similarly, no significant difference was found between participants with lower and higher educational attainment (U = 468.0, Z = -0.82, p = 0.412).

## Discussion

### Main findings

The findings from this study offer important insights into the factors influencing the acceptance of micronutrient therapy among patients with neurostress imbalances.

The high overall treatment acceptance, with a median score of 88.9, reflects a generally favorable response to the therapy, values underneath 50 being rare. This suggests that most participants perceived the treatment as acceptable, although individual responses varied widely, as indicated by the wide interquartile range. This variation may reflect differences in how participants evaluated the balance between perceived benefits and potential burdens or challenges associated with the treatment.

One of the key observations is the high acceptance of drug-related constraints (median score: 90), indicating that the practical aspects of the therapy, such as dosage and administration, were not seen as significant barriers by most participants. This aligns with existing research, which emphasizes that when patients find a treatment easy to integrate into their daily routines, they are more likely to adhere to it [24, 25]. In contrast, the comparatively lower acceptance of treatment duration (median score: 66.6) highlights a potential challenge in sustaining therapy over time. Although all respondents had been receiving treatment for at least three months at the time of the survey, information on longer treatment duration was not available, limiting conclusions regarding long-term acceptance. Nevertheless, even after several months of therapy, ongoing treatment may already impose psychological and practical constraints, which could contribute to greater ambivalence or reluctance [17–20]. These findings call for further evaluation of acceptance in relation to treatment duration.

The high acceptance of side effects (mean score: 90, median score: 100) is particularly encouraging. It suggests that participants either experienced few side effects or perceived them as minimal and manageable. This is consistent with the positive reception of the therapy’s efficacy (mean score: 83, median score: 100), reinforcing the idea that when side effects are mild, patients are more likely to perceive the treatment as beneficial [21, 22]. The strong correlation between general acceptance and perceived efficacy (r = 0.498, 95% CI [0.23, 0.69], p < 0.001) supports this conclusion, showing that patients who believe the therapy is effective are more likely to accept it. This finding is consistent with previous studies that highlight the importance of perceived efficacy in influencing treatment adherence [21, 22].

Interestingly, the correlation between general acceptance and compliance was not statistically significant (r = 0.216, 95% CI [-0.03, 0.44], p = 0.084), suggesting that factors beyond mere acceptance of the therapy may play a role in determining how closely patients follow treatment recommendations. This indicates that while acceptance is a key element, other factors such as lifestyle compatibility, personal motivation, or external support may also significantly influence adherence.

The association between general acceptance and the perceived lack of information was modest and imprecise (r = -0.248, 95% CI [-0.46, -0.01], p = 0.043), and should be interpreted with caution. This finding suggests that participants who reported greater informational deficits tended to report lower acceptance. This reinforces the importance of clear, comprehensive communication between healthcare providers and patients. Ensuring that patients fully understand the treatment’s purpose, potential side effects, and expected outcomes may boost acceptance and, potentially compliance.

Finally, the correlation between general acceptance and side effects (r = 0.401, 95% CI [0.18, 0.59], p < 0.001) is an important finding. It demonstrates that patients who experienced fewer side effects or perceived them as manageable were more likely to accept the therapy. This suggests that minimizing side effects or effectively managing patient expectations regarding side effects could significantly enhance treatment acceptance.

### Findings in context

The findings are consistent with previous research on treatment adherence in chronic conditions such as diabetes and hemophilia, where ease of integration into daily life and minimal side effects were found to promote patient acceptance and adherence. Sidani et al. (2009) highlighted that treatments are most likely to be accepted when they appear appropriate, efficient, and minimally disruptive criteria that seem largely met by micronutrient therapy in our cohort. The high tolerance for side effects observed in this study is in line with the favorable safety profile of micronutrient supplementation described in prior reports [13–15]. Conversely, the lower acceptance of treatment duration mirrors challenges documented in other therapeutic areas, where prolonged regimens often lead to decreased motivation and compliance over time [26]. Of particular interest is the negative correlation observed between acceptance and perceived lack of information. As higher scores on this item indicate greater informational deficits, this finding suggests that participants who felt less adequately informed reported lower acceptance. This may reflect a communication gap, whereby information was provided but not perceived as sufficient, clear, or appropriately tailored to patients’ needs. Alternatively, patients who felt well informed may have developed more realistic expectations and greater trust in the therapeutic approach, thereby reporting higher acceptance. Together, these observations emphasize that acceptance is a multidimensional construct shaped not only by therapy characteristics but also by patient perceptions, expectations, and the quality of patient-provider communication.

### Strengths and limitations

A major strength of this study is the use of the validated ACCEPT© questionnaire, which allows for a standardized and multidimensional assessment of treatment acceptance. In addition, the study population was broadly representative of the Swiss female population, which enhances generalizability.

However, several limitations should be acknowledged. An important limitation of this study relates to potential selection and response bias. Participants were contacted after having completed the neurostress profile assessment and having initiated individualized micronutrient therapy and were invited to complete an online questionnaire. As a result, respondents are likely enriched for patients who were more satisfied with the intervention, more adherent, or who perceived greater benefit. Conversely, individuals who discontinued treatment early, experienced limited benefit, or chose not to engage further with care may be underrepresented among respondents. This imbalance may have led to an overestimation of overall acceptance and perceived efficacy, as well as an underestimation of negative experiences. These findings should therefore be interpreted with caution and primarily viewed as reflective of patient perceptions among engaged and responding participants rather than the entire treated population.

Second, the data were collected through self-reported questionnaires, which may lead to an overestimation of actual adherence. This is particularly relevant due to the potential influence of social desirability bias, where participants might answer in ways that reflect more positively on their behavior, making them appear more accepting of the treatment than they truly are.

Third, the relatively short follow-up period restricts the ability to evaluate longer-term adherence and acceptance. Given that such treatments are often required over extended periods to fully address neurostress imbalances, the short time frame makes it challenging to predict how adherence may change over time, particularly as factors like treatment fatigue or evolving life circumstances come into play.

A further limitation of this work is the absence of objective clinical effectiveness outcomes. The study did not assess symptom changes, biological markers, or other clinical endpoints, and therefore cannot draw conclusions regarding therapeutic efficacy. Associations between acceptance and perceived efficacy should be interpreted as patient-reported perceptions rather than evidence of clinical benefit. However, perceived effectiveness is a recognized determinant of treatment acceptance and persistence, particularly in long-term or preventive interventions, and was therefore explored in this implementation-focused context.

### Implications and future research

The findings highlight the importance of patient-centered communication in optimizing therapy acceptance. Since general acceptance was closely linked to perceived efficacy and side effects, clinicians should focus on explaining realistic expectations regarding therapy benefits and addressing concerns about tolerability. Strategies aimed at supporting acceptance of ongoing therapy, such as individualized follow-up, motivational support, and shared decision-making, warrant further investigation. Studies with longer observation periods are needed to better characterize acceptance over time and its relationship with treatment persistence.

## Conclusion

In conclusion, this study suggests a high level of self-reported acceptance of individualized micronutrient therapy among responding participants in routine clinical practice. The findings highlight the complex relationships between treatment acceptance, perceived efficacy, perceived side effects, and patient-reported compliance. While overall acceptance was high, lower scores associated with perceived treatment duration and the influence of information provision indicate potential challenges to ongoing engagement. These results underscore the importance of patient education, expectation management, and consideration of treatment burden when implementing individualized micronutrient interventions. Future research should prospectively examine acceptance and treatment persistence over time, include non-responders and treatment discontinuers, and explore additional factors influencing engagement, such as lifestyle, contextual constraints, and patient motivation.

## Supplementary Information

Below is the link to the electronic supplementary material.

Supplementary file1 (PDF 122 KB)

Supplementary file2 (PDF 381 KB)

## Data Availability

No datasets were generated or analysed during the current study.
