# Supplementary material for: Patient acceptance and implementation of micronutrient therapy in women with neurostress-related symptoms
Source: Arch Gynecol Obstet. 2026 Feb 4;313(1):74. doi: 10.1007/s00404-026-08321-6 (PMC12872666; doi:10.1007/s00404-026-08321-6)
Supplement: Supplementary file 1 — Supplementary file1 (PDF 122 KB) [file 404_2026_8321_MOESM1_ESM.pdf]

Supplementary File 1: Full Survey Questionnaire, including the ACCEPT© Validated Questionnaire

1. Terms of Participation: Are you…
a. Of legal age

2. What symptoms did you have before starting micronutrient therapy? (Multiple answers possible)
a. Depressive mood
b. Anxiety / Inner restlessness
c. Irritability / Aggressiveness
d. Sleep disturbance
e. Lack of drive
f. "Cotton wool in the head" / Headaches
g. Forgetfulness / Difficulty concentrating
h. Fatigue / Exhaustion
i. Disordered eating regulation / Digestive disturbances
j. Reduced regeneration capacity / Loss of vitality
k. Low libido
l. Impaired stress management / Feeling overwhelmed
m. Other (Text box)
Scale of 1-4 (not at all to very strong) for each symptom

3. Have you already tried other therapies or medications for the symptoms mentioned above?
a. Yes
b. No
If Yes:
4. How many? (Text box)

5. For what period did you take/use these therapies?
a. < 1 month
b. 1-6 months
c. 6-12 months
d. 1-2 years
e. > 2 years

6. Were these therapies effective for you?
a. Yes
b. No

7. Where did you first hear about the neurostress profile?
a. General practitioner
b. Gynecologist
c. Therapist
d. Internet
e. Friends/Family

f. Never heard of it
g. Other (Text box)

8. Were the information you received about your diagnosis (neurostress profile) clear to you?
a. The information was very clear
b. The information was sufficiently clear
c. The information was not sufficiently clear
d. The information was not clear at all

9. Do you feel well-informed about the goal of micronutrient therapy?
a. I feel very well informed
b. I feel sufficiently informed
c. I do not feel sufficiently informed
d. I do not feel informed at all

10. Have you taken the micronutrients on average at least 6 days per week since the start of the therapy?
a. Yes
b. No

Validated ACCEPT© Questionnaire

11. Do you find the preparation of your medication cumbersome?
a. Yes, and I find it hard to accept
b. Yes, but I find it easy to accept
c. No
d. My medication does not require preparation

12. Do you find taking the medication cumbersome?
(Examples of medication administration: swallowing, injection with a syringe, inhalation through the nose, etc.)
a. Yes, and I find it hard to accept
b. Yes, but I find it easy to accept
c. No

13. Do you find the form of your medication unpleasant?
(Examples of medication forms: tablet, capsule, powder in packets, injection, drops, inhaler, etc.)
a. No
b. Yes, and I find it hard to accept
c. Yes, but I find it easy to accept

14. Have you been taking your medication for a long time?
a. Yes, and I find it hard to accept
b. Yes, but I find it easy to accept
c. No

15. Will you have to take your medication for a long time?
a. No
b. Yes, and I find it hard to accept
c. Yes, but I find it easy to accept

16. Do you feel restricted by having to remember to take your medication?
a. No
b. Yes, and I find it hard to accept
c. Yes, but I find it easy to accept

17. Do you feel restricted by having to take the time to pick up your medication from the pharmacy?
a. Yes, and I find it hard to accept
b. Yes, but I find it easy to accept
c. No

18. Do you feel restricted by having to remember to bring your medication with you?
a. No
b. Yes, and I find it hard to accept
c. Yes, but I find it easy to accept

19. Do you feel restricted by always having to carry your medication with you?
a. Yes, and I find it hard to accept
b. Yes, but I find it easy to accept
c. No
d. I never need to carry my medication
20. Does your medication need to be stored under special conditions while traveling?
a. No
b. Yes, and I find it hard to accept
c. Yes, but I find it easy to accept

21. Do you feel you take many medications?
a. Yes, and I find it hard to accept
b. Yes, but I find it easy to accept
c. No

22. Can you take your medication discreetly?
a. Yes
b. No, and I find it hard to accept
c. No, but I find it easy to accept

23. Do you find that regularly taking your medication has become part of your normal daily routine?
a. Yes
b. No, and I find it hard to accept
c. No, but I find it easy to accept
d. I do not need to take my medication regularly

24. Do you feel restricted by how often you need to take your medication?
a. No
b. Yes, and I find it hard to accept
c. Yes, but I find it easy to accept

25. Does your medication have side effects?
a. Yes, and I find it hard to accept
b. Yes, but I find it easy to accept
c. No

26. Are these side effects unpleasant?
a. Yes, and I find it hard to accept
b. Yes, but I find it easy to accept
c. No
d. I have no side effects

27. Are you affected by these side effects?
a. Yes, and I find it hard to accept
b. Yes, but I find it easy to accept
c. No
d. I have no side effects

28. Do you need to take additional medication for the side effects of your medication?
a. Yes, and I find it hard to accept
b. Yes, but I find it easy to accept
c. No
d. I have no side effects

29. Is there a risk of serious side effects from your medication?
a. Yes, and I find it hard to accept
b. Yes, but I find it easy to accept
c. No
d. I do not know

30. Do you think your medication works for you?
a. No, and I find it hard to accept
b. No, but I find it easy to accept
c. Yes
d. I do not know

31. Do you think your medication provides sufficient protection?
a. No, and I find it hard to accept
b. No, but I find it easy to accept
c. Yes
d. I do not know
e. My medication is not meant to protect me

32. Does your medication have a fast effect on your condition?
a. Yes
b. No, and I find it hard to accept
c. No, but I find it easy to accept

33. Do you agree with the following statement: "My medication has more benefits than drawbacks."
a. Strongly disagree
b. Disagree
c. Agree somewhat
d. Strongly agree
e. I do not know

34. When you consider the benefits and drawbacks of your medication, do you find it an acceptable solution?
a. Not acceptable at all
b. Not very acceptable
c. Somewhat acceptable
d. Fully acceptable
e. I do not know

35. Are you convinced that it is worth taking your medication in the long run?
a. Not at all convinced
b. Not really convinced

c. Somewhat convinced
d. Fully convinced
e. I do not know

Supplementary Questions and Demographics

36. Do you currently still have any symptoms?
a. Yes
b. No
If Yes:

37. What symptoms do you currently have? (Multiple answers possible)
a. Depressive mood
b. Anxiety / Inner restlessness
c. Irritability / Aggressiveness
d. Sleep disturbance
e. Lack of drive
f. "Cotton wool in the head" / Headaches
g. Forgetfulness / Difficulty concentrating
h. Fatigue / Exhaustion
i. Disordered eating regulation / Digestive disturbances
j. Reduced regeneration capacity / Loss of vitality
k. Low libido
l. Impaired stress management / Feeling overwhelmed
m. Other (Text box)
Scale of 1-4 (not at all to very strong) for each symptom

38. Would you recommend micronutrient therapy?
a. Yes
b. Probably yes
c. Probably no
d. No

39. What is your highest level of education?
a. No school leaving qualification
b. Basic/secondary school diploma
c. High school / Vocational school (FMS) / Vocational school (BMS)
d. Completed apprenticeship
e. Bachelor
f. Master
g. Doctorate / Habilitation

40. Which of the following categories best describes your employment status?
a. 1-41 hours per week (part-time)
b. 42 hours per week or more (full-time)
c. Unemployed and seeking work
d. Unemployed and not seeking work
e. AHV
f. IV

42. What is your monthly net income?
a. < 5,000 CHF
b. 5,000 – 10,000 CHF

c. > 10,000 CHF
d. I have no income

43. What is your current marital status?
a. Single
b. In a registered partnership
c. Married
d. Divorced
e. Widowed
