# Supplementary material for: Patient acceptance and implementation of micronutrient therapy in women with neurostress-related symptoms
Source: Arch Gynecol Obstet. 2026 Feb 4;313(1):74. doi: 10.1007/s00404-026-08321-6 (PMC12872666; doi:10.1007/s00404-026-08321-6)
Supplement: Supplementary file 2 — Supplementary file2 (PDF 381 KB) [file 404_2026_8321_MOESM2_ESM.pdf]

Kanton Bern
Canton de Berne

Gesundheits-, Sozial- und Integrationsdirektion
Kantonale Ethikkommission für die Forschung

Murtenstrasse 31
3010 Bern
Bern
+41 31 633 70 70 (Telefon)
+41 31 633 70 71 (Telefax)
info.kek.kapa@be.ch
www.be.ch/gsi

Dorothy Pfiffner
+41 31 633 70 77
dorothy.pfiffner@be.ch

GSI-KEK, Murtenstrasse 31, 3010 Bern

Frau
Prof. Dr. med. Petra Stute
Universitätsklinik für Frauenklinik
Endokrinologie und Reproduktionsmedizin
Inselspital Bern
Friedbühlstrasse 19
3010 Bern

Bern, 09. August 2021, FN

Bewilligung der KEK Bern

**Project-ID**	2021-01019
**Projekttitel**	Akzeptanz und Empfehlungsumsetzung des Neurostressprofils
**Master-/Doktorarbeit von**	Loviat, Fanette
**Projektleitung**	Prof. Dr. med. Petra Stute
**Sponsor**	Prof. Dr. med. Petra Stute
**Zentren**	• Prof. Dr. med. Petra Stute, Universitätsklinik für Frauenklinik, Inselspital, Bern

Entscheid

☒ Die Bewilligung wird erteilt

Klassifizierung

☒ Forschungsprojekt gemäss HFV, Kategorie: A
- ☒ Forschung mit Personen
- ☐ Weiterverwendung des biologischen Materials oder der gesundheitsbezogenen Personendaten
- ☐ mit Verstorbenen
- ☐ mit Embryonen / Föten
- ☐ mit ionisierender Strahlung

Entscheidverfahren

☐ ordentliches Verfahren ☐ vereinfachtes Verfahren ☒ Präsidialverfahren

Die Ethikkommission bestätigt, dass sie nach ICH-GCP arbeitet.

Gebühren

Betrag: CHF 0.– **Tarifcode:**

Gemäss der geltenden Gebührenordnung von swissethics. Rechnungsstellung folgt durch die Kantonale Gesundheits-, Sozial- und Integrationsdirektion (GSI).

Rechtsmittelbelehrung

Gegen diese Verfügung kann innert 30 Tagen seit Eröffnung bei der Gesundheits-, Sozial-und Integrationsdirektion des Kantons Bern Beschwerde erhoben werden. Die Beschwerdefrist kann nicht verlängert werden. Die Beschwerdeschrift ist im Doppel bei der Gesundheits-, Sozial-und Integrationsdirektion, Rathausgasse 1, Postfach, 3000 Bern 8 einzureichen.

Eine allfällige Beschwerde, die in mindestens zwei Exemplaren einzureichen ist, muss einen Antrag, die Angabe von Tatsachen und Beweismitteln, eine Begründung sowie eine Unterschrift enthalten; der angefochtene Entscheid und andere greifbare Beweismittel sind beizulegen.

Sie muss

(a) angeben, welche Entscheidung anstelle der angefochtenen Verfügung beantragt wird und
(b) aus welchen Gründen diese andere Entscheidung verlangt wird sowie
(c) die Unterschrift der beschwerdeführenden Partei oder der sie vertretenden Person enthalten.

Der Beschwerdeschrift beizulegen sind die Beweismittel, soweit sie greifbar sind, und die angefochtene Verfügung. (Art. 32 und 60 ff. des Gesetzes vom 23. Mai 1989 über die Verwaltungsrechtspflege [VRPG; BSG 155.21]).

Kopie an

☐ BAG

☒ DLF

☐ Andere

Prof. Dr. med. Christian Seiler
Präsident KEK Bern

Dr. sc. nat. Dorothy Pfiffner
Vizepräsidentin
Leiterin Wissenschaftliches Sekretariat

Anhang: -Pflichten des Sponsors/der Prüfperson oder der Projektleitung
-Mögliche Entscheide und ihre Bedeutung
-Eingereichte Dokumente, Stand vom 06.08.2021

Anhang

Pflichten des Sponsors/der Projektleitung

Einreichung Dokumente: revidierte Dokumente und neue Dokumente zur Studie/zum Projekt sollen ausschliesslich über das Web-Portal BASEC eingereicht werden, auf der entsprechenden Formularseite des betreffenden Gesuches. Obsolete Dokumente sind dabei zu entfernen und Datums- und Versionsangaben entsprechend zu ergänzen. Die erfolgten Änderungen müssen im Korrekturmodus abgefasst werden und zusätzlich als ‚clean'-Version eingereicht werden. Die Studieninformationen und -einwilligungen, das Protokoll und die Amendments müssen in durchsuchbaren PDF-Dateien eingereicht werden, insbesondere müssen gescannte Dokumente eine Texterkennung durchlaufen haben (OCR). Das unterschriebene und datierte Begleitschreiben muss die Antworten auf eventuell von der EK gestellte Fragen enthalten. Revidierte Dokumente sind auch den weiteren Zulassungsbehörden zuzustellen, sofern diese involviert sind.

Anmerkung: Die zuständige Ethikkommission überprüft im Rahmen des Bewilligungsverfahrens Aufklärungsbogen und Einwilligungserklärung in einer der Amtssprachen Deutsch, Französisch oder Italienisch. Aufklärungsbogen und Einwilligungserklärung in einer anderen Sprache werden von der Ethikkommission lediglich zur Kenntnis genommen. Für die korrekte Übersetzung ist der Sponsor oder die Projektleitung verantwortlich.

Meldepflichten: Die rechtlich bindenden Melde- resp. Bewilligungspflichten an die Ethikkommission für wesentliche Änderungen, einen vorzeitigen Studienabbruch, unerwünschte Ereignisse u.a. sind einzuhalten (Verordnungen des Bundes). Der Abschlussbericht ist spätesten ein Jahr nach Studienende der Ethikkommission einzureichen.

Registrierungspflicht: Der Sponsor muss – falls es sich um einen klinischen Versuch handelt – diesen in einem WHO-Primärregister oder im Register der Nationalen Medizinbibliothek der USA (clinicaltrials.gov) erfassen und anschliessend diese Nummer im BASEC-Portal eingeben. Die Übertragung der erforderlichen Daten in das Swiss National Clinical Trials Portal (SNCTP) kann nach Bewilligung der Ethikkommission und Zustimmung des Gesuchstellers automatisch erfolgen. Die Informationen über den klinischen Versuch sind in beiden Registern öffentlich zugänglich. Zusätzlich veröffentlicht swissethics wenige Informationen wie Titel, Projekttyp oder Leit-Ethikkommission aller durch die kantonalen Ethikkommissionen bewilligten Gesuche auf swissethics.ch (ausser Phase-I-Studien).

Mögliche Entscheide und ihre Bedeutung

Die Bewilligung wird erteilt: Das Vorhaben kann gemäss bewilligtem Forschungsplan und im Rahmen der anwendbaren rechtlichen Bestimmungen durchgeführt werden. Weitere Bewilligungspflichten (Swissmedic/BAG) sind zu beachten

Eingereichte Dokumente für das Hauptzentrum

Prof. Dr. med. Petra Stute, Universitätsklinik für Frauenklinik, Inselspital, Bern		
Dokument	Dok.Datum	Version
**1. Cover Letter**		
28-04-2021-coverletter-fl-neurostress.pdf	28/04/2021	
**3. Participant information sheet and informed consent (ICF)**		
studieninformation-neurostress-neu.docx	28/06/2021	3
**4. Study plan (protocol), signed and dated**		
studyprotocol-neurostress-neu-2.pdf	28/06/2021	3
studyprotocol-neurostress-neu.docx	28/06/2021	3
**6. Investigator's CV, dated**		
cv-p-stute-en-sig.pdf	12/06/2020	
**11. Other documents handed over to study participants**		
fragebogen-fl-neurostress.pdf	14/04/2021	
**12. Details on nature and scope/value of compensation for participants**		
There is no compensation for the participation in this study		
**14. Information on secure handling of biological material and personal data, and in particular on the storage thereof**		
see doc/cat: **4**, page/ref: **11**		
**39. Miscellaneous / Varia**		
masterarbeitsbestatigung.png	18/07/2021	1
